# Evaluation of Mucosamin Effect on Treating Radiation Induced Oral Mucositis during and after Radiotherapy amongst Patients with Oral Cavity Squamous Cell Carcinoma

**DOI:** 10.31557/APJCP.2021.22.11.3711

**Published:** 2021-11

**Authors:** Hamid Nasrollahi, Saeedeh Khaki, Mansour Ansari, Niloofar Ahmadloo, Nezhat Khanjani, Sayed Hasan Hamedi, Shapoor Omidvari, Ahmad Mosalaei, Mohammad Mohammadianpanah, Behnam Kadkhodaei

**Affiliations:** 1 *Department of Radiation Oncology, Nemazee Hospital, Shiraz University of Medical Sciences, Shiraz, Iran. *; 2 *Breast Diseases Research Center, Shiraz University of Medical Sciences Shiraz, Iran. *; 3 *Department of Health Administration, School of Management and Information Sciences, Shiraz University of Medical Sciences, Shiraz, Iran. *; 4 *Shiraz Institute for Cancer Research, Shiraz University of Medical Sciences, Shiraz, Iran.*; 5 *Colorectal Research Center, Zand, Shiraz, Iran. *

**Keywords:** Oral cavity squamous cell carcinoma, radiotherapy, oral mucositis, mucosamin

## Abstract

**Background:**

Oral mucositis is a serious complication radiation therapy for cancer. This is a major complication during radiation therapy of the head and neck tumors in approximately all patients. Therefore, this study was conducted to evaluate the effect of Mucosamin on treatment of radiation induced oral mucositis during and after radiotherapy amongst patients with oral cavity squamous cell carcinoma.

**Materials and Methods:**

In this prospective clinical trial, eligible patients who referred to radiation oncology department of Namazi Hospital, Shiraz, Iran from Jan 2018 till Jan 2019 were evaluated. The cases with confirmed pathologic diagnosis of squamous cell carcinoma of the oral cavity underwent 6,000 cGy radiation therapy and were randomly divided into two groups: 1- Intervention group; Mucosamin spray for 3-4 times a day (n = 40); 2 - Control group; standard medications (3 times a day) (n = 40). Oral mucositis was evaluated weekly based on RTOG scoring scale. Grade of mucositis was recorded during treatment and after radiation therapy.

**Results:**

A total of 80 patients were divided in two groups of Mucosamin and control. From week 3 until the end of radiotherapy (week 6) and after radiotherapy (week 8), there was a significant difference in the severity of oral mucositis between the Mucosamin and the control groups (P <0.05).

**Conclusion:**

The results of this study showed that Mucosamin spray was able to significantly improved radiation-induced oral mucositis in patients with oral squamous cell carcinoma.

## Introduction

Oral cavity cancers account for 2-4 percent of all human cancers. In some areas of the world, the incidence of oral cancer is higher; for example, in India and Pakistan, 45% and 10% of all cancers are Oral squamous cell carcinoma (OSCC) (Sharma et al., 2010). From 2004 till 2009, more than 300,000 new cases of oral cancer were diagnosed worldwide. The OSCC is the most common oral tumor. Studies showed that OSCC accounts for more than 90% of all oral cavity cancers (Markopoulos, 2012).

Radiation therapy (RT) is an important part of OSCC treatment (Kwong, 2004) ; however, oral mucositis is a serious complication during radiotherapy. This is a major complication during radiation therapy for head and neck tumors that occurs in all of the patients. Mucosal damage appears gradually, and it takes a long time to become visible. The peak of oral mucositis is about 7 to 9 weeks after the initiation of radiotherapy and heals 2-10 weeks after the end of cancer treatment (Pauloski, 2011). The severity of mucositis induced by radiation is strongly dependent on dose, fraction size, radiation portal, segmentation, and type of ionized radiation. The most important risk factors for oral mucositis are: poor oral hygiene and periodontal disease (Khaw et al., 2014), chronic alcohol usage, smoking (Riley et al .,2015), hypolysis (Jensen and Vissink 2014), low body mass index (BMI <18.5) and diabetes (Tao et al., 2017). Oral mucositis is associated with other problems, including oral cavity discomfort, pain, malnutrition, delayed drug control, increased hospitalization costs, and threatening infection in some patients; therefore, it should be prevented and limited as much as possible (Agarwal, 2012).

Treatment modalities for radiation-induced oral mucositis include supportive care, ice chips, aloe vera, honey and gastric tubes to improve nutritional condition (Moslemi et al., 2016). Many clinical trials have published protocols for the prevention or treatment of mucositis, but there is still no general agreement (De Sanctis et al., 2016). Recently, it was suggested that compounds containing amino acids and sodium hyaluronate lead to faster healing and pain control of the oral mucositis (Colella et al., 2012). Evidence has shown that the use of external hyaluronic acid can stimulate and accelerate wound healing by inducing the production of growth factors, different types of collagen, and proliferation of fibroblasts (Romeo et al., 2014).

Given the importance of controlling and treating radiotherapy complications in patients with oral cavity cancer, this study was performed to evaluate the effect of mucosamin in the treatment of oral cavity mucositis induced by radiation therapy during and after radiation therapy.

## Materials and Methods

In this phase 3 clinical trial , patients with OSCC cancer who referred to the radiotherapy and oncology department of Namazi Hospital, Shiraz, Iran were enrolled in this study. Inclusion criteria were people aged 18-75 with oral cancers that were confirmed by histologic diagnosis and functional status based on the Eastern Community Oncology Group (ECOG) criterion of less than or equal to 2. Exclusion criteria included patients with systemic disease affecting wound healing (such as diabetes mellitus or collagen vascular diseases), mucosal and skin diseases, poor oral health infection, active gingivitis or ulceration of the mouth, previous head and neck surgery, radiotherapy and chemotherapy due to malignancy or distant metastases at the time of diagnosis. According to previous studies, 80 patients were recruited (Charalambous et al., 2018; Ruggiero et al., 2016; Mosalaei et al., 2010).

Initial evaluation included history and physical examination. Checklists included demographic information (age, gender), complete medical and surgical history, physical examination and clinical condition of the patient.

Patients with OSCC received at least 6000 cGy in the oral cavity. The participants were randomly assigned into 2 groups, intervention and the control group. Patients in the intervention group received mucosamin spray 3-4 times daily (n = 40) and the control group received standard drugs (magnesium, aluminum hydroxide, diphenhydramine and nystatin 3 times) (n = 40). In this medical department, standard treatment is a mixture of 5 cc (1 spoon) diphenhydramine, 5 cc Al-Mg suspension and 10 drops of nystatin mixed in a cup, and then gargled and swallowed. Oral mucositis was evaluated weekly according to RTOG scoring criteria. (table1) (Cox et al., 1995).

Mucosamin spray was used 3-4 times a day, each time 2-3 puffs. Treatment with mucosamin was initiated and continued 4 days before RT and continued up until the time that the oral lesions were healed completly.

Mucosamin spray was prepared by Arena Life Science Co and its agent in Iran, Elit Daru. Active ingredients of mucosamin spray are Sodium Hyaluronate, Glycine, L-Leucine, L-Lysine. Each bottle contained 30 ml which was given to patients free of charge. 

Mucositis was recorded by a radiation oncologist during treatment and in the first and second weeks after radiation therapy. Since there is no standard treatment for oral mucositis, treatments would be merely supportive therapy.

## Results

A total of 80 patients with OSCC were evaluated for the effects of mucosamin on radiation-induced oral mucositis in the intervention and control groups (table 2). The mean age of the patients in the mucosamin group was 54.22±10.22 (median 57 and range 28-70) and in the control group was 58.32±14.28 (median 64.5 and range 19-72). In the mucosamin group, there were 24 (60.0%) males and 16 (40.0%) females, and in the control group there were 18 (45.0%) males and 22 (55.0%) females.

The anatomic site of the tumor in all patients was oral tongue 78 (97.5%), the mandibular in one (1.3%) and the retro-mandibular trigone in one (1.3%). All patients (100%, 40 patients) in mucosamin group had oral tongue SCC and in the control group 38 (95%) had oral tongue SCC and in two patients’ cancer was in non-tongue area (5%).

With respect to the stage of the disease, 13 patients (32.5%) Stage I, 16 (40.0%) Stage II and 11 (27.5%) Stage III were in the mucosamin group (Figure 2-4). In the control group, 16 (40.0%) patients were in Stage I, 10 (25.0%) were in Stage II and 14 (35.0%) were in Stage III disease.

Patients received at least 60 Gy in oral cavity. In the control group the mean dose was 64.25±3.98 Gy and in the mucosamin group it was 65.10±3.73 Gy, but the difference was not statistically significant.

In the chemotherapy or Target therapy, in the mucosamin group, 20 patients (0.50%) received the chemo-radiotherapy (CRT) and 20 patients (0.50%) did not receive the CRT, and in the control group 21 (52.5%) patients received CRT and 19 (47.5%) patients did not receive the CRT. 

Regarding the regimen used in those were receiving chemotherapy or Target therapy, in the mucosamin group, 15 (37.5%) patients received cetuximab and 5 (12.50%) patients received cisplatin, and in the control group, 18 (85.7%) patients received cetuximab and 3 (14.3%) patients received cisplatin. None of the above factors, including age, anatomic site, stage, chemotherapy or targeted therapy were significantly different between the 2 groups. The mean RT dose was also similar in both groups.

 As shown in table (3), mucositis was significantly more severe in the control group in comparison to the mucosamin group from week 3 until the end of radiation therapy (week 6) and afterwards (week 8).

By comparing the duration of symptoms for oral mucositis in patients with squamous cell carcinoma of the oral cavity under radiotherapy showed that the mean duration of symptoms in the mucosamin group was 3.65 ±1.09 weeks (median 3 and range 1-6), and in the control group it was 5.22±1.07 weeks(median 5, range 1-6).

Amongst the patients, 2 (0.5%) in the mucosamin group and 4 (10.0%) in the control group stopped the treatment in less than one week. One person (2.5%) in the mucosamin group and 2 patients (5%) in the control group were admitted to the hospital following severe clinical and general condition; however, returned to the study with improvement and were included in the final analysis.

**Table 1:** RTOG Scoring Criteria for Oral Mucositis

Grade	1	2	3	4
Mucus membrane	Irritation/may experience mild pain not requiring analgesic	Patchy mucositis that may produce an inflammatory serosanguinous discharge/may experience moderate pain requiring analgesia	Confluent fibrinous mucositis/may include severe pain requiring narcotic	Ulceration, hemorrhage or necrosis

**Table 2:** Patients Demographic Information in Both Groups

		Control group	Mucosamin group	p-value
Mean age		58.32 ± 14.2	54.22 ± 10.22	0.144
				
Gender	Male	18 (45%)	24 (60%)	0.263
	Female	22 (55%)	16 (40%)	
Stage	1	16 (40%)	13 (32.5%)	0.358
	2	10 (25%)	16 (40%)	
	3	14 (35%)	11 (27.55)	
Anatomical site	Tongue	38 (95%)	40 (100%)	
	Other	2 (5%)	0 (0%)	
Concurrent	Yes	21 (52.5%)	20 (50%)	0.999
	NO	19 (47.5%)	20 (50%)	
Chemotherapy		3 (14.3%)	5 (25%)	0.454
Immunotherapy		18 (85.7%)	15 (75%)	

**Table 3:** severity of Mucositis in Mucosamin and Control Group during Treatment

Severity of Mucositis// Time	Group										
	Mucosamin (n = 40)					Control (n = 40)					P value
	Grade 0	Grade 1	Garde 2	Grade 3	Grade 4	Grade 0	Grade 1	Garde 2	Grade 3	Grade 4	
	n (%)	n (%)	n (%)	n (%)	n (%)	n (%)	n (%)	n (%)	n (%)	n (%)	
Week 1	39 (97.5)	1 (2.5)	0	0	0	33 (82.5)	7 (17.5)	0	0	0	0.057
Week 2	5 (12.5)	33 (82.5)	1 (2.5)	1 (2.5)	1 (2.5)	0	39 (97.5)	25 (62.5)	0	0	0.09
Week 3	0	28 (70.0)	12 (30.0)	0	0	0	15 (37.5)	25 (62.5)	0	0	0.007
Week 4	0	9 (22.5)	30 (75.0)	1 (2.5)	0	0	0	38 (95.0)	2 (5.0)	0	0.006
Week 5	0	1 (2.5)	36 (90.0)	2 (5.0)	1 (2.5)	0	0	21 (52.5)	19 (47.5)	0	< 0.001
Week 6	0	0	34 (85.0)	6 (15.0)	0	0	0	18 (45.0)	20 (50.0)	2 (5.0)	0.001
Week 7	0	20 (50.0)	17 (47.5)	3 (7.5)	0	0	2 (5.0)	26 (65.0)	10 (25.0)	2 (5.0)	< 0.001
Week 8	1 (2.5)	33 (82.5)	6 (15.0)	0	0	0	19 (47.5)	15 (37.5)	6 (15.0)	0	0.002

## Discussion

 In the present study, patients diagnosed with squamous cell carcinoma of the oral cavity received 60 Gy radiotherapy over a period of 6 weeks, some received chemotherapy or target therapy. The mucositis was evaluated and the effects of mucosamin spray on the improvement and reduction of these complications as well as duration of symptoms were compared with the control group. The two groups were matched for age, gender, anatomical location of the lesion, and stage of the disease, rate of chemotherapy or Target therapy, and type of medication used.

The results showed that mucosamin spray was able to significantly reduce the severity of oral mucositis during and after radiotherapy, so that the oral mucositis grade (according to RTOG criteria) during weeks 3 to 8 (during and after radiotherapy) showed a significant reduction in comparison with the control group. 

Few studies were performed on the effects of mucosamin on radiation-induced oral mucositis in patients with head and neck cancer. Other researchers have done similar studies in which the effects of other medicinal or herbal compounds on improving head and neck radiotherapy complications were investigated.

Clémenson (2019) studied the preventive effects of topical amifostine-derived thermogels and showed that the use of this thermogel prior to radiation significantly reduced the severity of oral mucositis as well as dermatitis induced radiotherapy. It reduces the viability of the murine model and at the same time, does not diminish the efficacy of radiation therapy against sub-mucosal layer tumors and subcutaneous areas (Clémenson et al., 2019). In this study, we evaluated the effects of mucosamin on radiation-induced oral mucositis in the head and neck region and achieved similar results to those found in Charalambous (2018) who investigated the effect of thyme honey on radiation-induced oral mucositis. In their study they found that in patients with head and neck cancer, in the intervention group showed milder degrees of oral mucositis during evaluations, and the quality of life in the intervention group was significantly higher than in the control group (Charalambous et al., 2018). Although in the present study drug intervention significantly improved oral mucositis, we did not examine the quality of life of patients. 

The results of Rugierro (2016) study on the potential effect of mucosamin for the treatment of symptomatic oral mucositis in patients undergoing hematopoietic stem cell transplantation showed that in patients treated with mucosamin, severe OM cases occurred less than the control group; hence, mucosamin might have protective role against severe mucositis phases (Ruggiero et al., 2016), which is consistent with the findings of the present study, although the type of cancer and organ involved in the two studies are different.

 Collela (2012) examined the effect of spray containing hyaluronate Sodium and a set of collagen precursor amino acids on radiation-induced OM. Lesions of patients treated with sodium hyaluronate- amino-acids (SH-AA) spray also improved significantly after 72 hours of treatment (Colella et al., 2012). In the present study, mucosamin spray, similar to sodium hyaluronate, significantly reduced oral mucositis and its severity; in addition the duration of symptom was significantly shorter compared to the control group. Mucosamin spray contains Sodium Hyaluronate, Glycine, L-Leucine, L-Lysine 

A study by Mosalaei (2010) on the effect of zinc sulfate on the prevention and treatment of oral mucositis induced by radiotherapy, showed a reduction in the severity of mucositis in the zinc group, but the time of mucositis onset was not different between the two groups (Mosalaei et al., 2010). In the present study, there was a significant decrease in the symptoms of oral mucositis amongst patients in the intervention group and these effects were significant between weeks 3 and 6 and up to 2 weeks after the end of radiation.

Despite the high incidence of radiation-induced oral mucositis, this complication is usually self-limiting. Many clinical studies were conducted to prevent and treat radiation-induced oral mucositis; however, no single drug or treatment regimen as a standard treatment can significantly improve radiation-induced oral mucositis. 

## Author Contribution Statement

None.
